# Targeted Therapy against Metastatic Melanoma Based on Self‐Assembled Metal‐Phenolic Nanocomplexes Comprised of Green Tea Catechin

**DOI:** 10.1002/advs.201801688

**Published:** 2019-01-15

**Authors:** Ke Li, Gao Xiao, Joseph J. Richardson, Blaise L. Tardy, Hirotaka Ejima, Wen Huang, Junling Guo, Xuepin Liao, Bi Shi

**Affiliations:** ^1^ Department of Biomass Chemistry and Engineering Sichuan University Chengdu 610065 China; ^2^ Laboratory of Ethnopharmacology Regenerative Medicine Research Center West China Hospital Sichuan University Chengdu Sichuan 610041 China; ^3^ Wyss Institute for Biologically Inspired Engineering John A. Paulson School of Engineering and Applied Sciences Harvard University Boston MA 02115 USA; ^4^ Department of Environmental Science and Engineering College of Environment and Resources Fuzhou University Fuzhou 350108 China; ^5^ ARC Centre of Excellence in Convergent Bio‐Nano Science and Technology and Department of Chemical and Biomolecular Engineering The University of Melbourne Parkville Victoria 3010 Australia; ^6^ Department of Bioproducts and Biosystems School of Chemical Engineering Aalto University P. O. Box 16300 00076 Finland; ^7^ Department of Materials Engineering The University of Tokyo 7‐3‐1 Hongo Bunkyo‐ku Tokyo 113‐8656 Japan; ^8^ National Engineering Laboratory for Clean Technology of Leather Manufacture Sichuan University Chengdu Sichuan 610065 China

**Keywords:** metal‐phenolic network, metastatic melanoma, polyphenols, self‐assembly, targeted therapy

## Abstract

The targeted therapy of metastatic melanoma is an important yet challenging goal that has received only limited attention to date. Herein, green tea polyphenols, (–)‐epigallocatechin‐3‐gallate (EGCG), and lanthanide metal ions (Sm^3+^) are used as building blocks to engineer self‐assembled Sm^III^‐EGCG nanocomplexes with synergistically enhanced tumor inhibitory properties. These nanocomplexes have negligible systemic toxic effects on healthy cells but cause a significant reduction in the viability of melanoma cells by efficiently regulating their metabolic pathways. Moreover, the wound‐induced migration of melanoma cells can be efficiently inhibited by Sm^III^‐EGCG, which is a key criterion for metastatic melanoma therapy. In a mouse melanoma tumor model, Sm^III^‐EGCG is directly compared with a clinical anticancer drug, 5‐fluorouracil and shows remarkable tumor inhibition. Moreover, the targeted therapy of Sm^III^‐EGCG is shown to prevent metastatic lung melanoma from spreading to main organs with no adverse side effects on the body weight or organs. These in vivo results demonstrate significant advantages of Sm^III^‐EGCG over its clinical counterpart. The results suggest that these green tea‐based, self‐assembled nanocomplexes possess all of the key traits of a clinically promising candidate to address the challenges associated with the treatment of advanced stage metastatic melanoma.

Cutaneous melanoma is one of the most lethal and fastest growing forms of human cancer, tending to affect a younger population when compared with other cancers.^1^ For example, in the United States melanoma is one of the most common forms of skin cancer, with 76 380 new cases estimated in 2016.^2^ Early‐stage melanoma is curable with a success rate of 98% through surgical resection; however, advanced stage metastasis results in poor prognosis, with five‐year survival rates dramatically dropping to 17%.^3^ Moreover, early melanoma detection is hindered by the lack of appropriate tumor biomarkers and inadequate public education. Moreover, with an absence of clinically significant symptoms, the early melanoma can reach an advanced stage aggressively with no attention.^4^ Therefore, the success rate for the treatment of melanoma is relatively low compared with other cancer types. Despite ongoing advancement in the study of melanoma, including surgical excision, radiation therapy, immunotherapy, and chemotherapy, the available treatment options are much more limited for metastatic stage patients because metastatic melanoma is noted for its high drug resistance, uncontrolled proliferation, and distant metastases.^5^ For example, surgical resections with preoperative serine/threonine‐protein kinase B‐Raf (BRAF) inhibitors (the first‐line therapy for melanoma treatment) or interleukin‐2 biological therapies have different shortcomings such as toxicities, unsatisfactory efficacy, and rapid development of resistance, thus significantly limiting their long‐term therapeutic effects.^3^

Targeted drug delivery systems that can directly deliver drugs to a specific site with minimal systemic exposure provide significant advantages over current treatments;^6^, ^7^, ^8^, ^9^, ^10^, ^11^, ^12^ however, the bioavailability of therapeutic molecules delivered through drug carriers targeted to metastatic melanoma remains low. This is mainly due to the rapid proliferation and bloodstream/lymphatic migration of metastatic melanoma. Moreover, due to the high mobility of metastatic melanoma, the tumors are generally highly dispersed into a large number of spreading nodules without the typical molecular and fluid transport dynamics generated by other types of tumors.^13^, ^14^ Therefore, the proposed mechanism of many carrier‐based targeted therapies has a low efficiency for metastatic melanoma.^15^ As a result, these carrier‐drug composites still require high doses and systemic administration, which increase their cost as well as their side effects. These numerous observations highlight the critical need to develop a novel therapeutic platform that can provide accurate cellular targeting towards spreading melanoma cells with low off‐target effects.^16^, ^17^, ^18^

A wide variety of drug carriers have been developed to enhance the pharmacokinetic performance and biodistribution of drugs; however, the carrier is generally just an excipient for delivery goal where only the drug molecule is the therapeutically relevant compound.^19^, ^20^, ^21^, ^22^, ^23^, ^24^, ^25^ Natural compound‐based nanoparticles are a minimally explored area of drug delivery with significant promise for cancer therapy. Research into natural compound‐based nanocarriers as cancer therapies has largely focused on using them as a delivery platform for conventional chemotherapeutics.^26^, ^27^ The tendency of such nanosystems to preferentially interact with and to be ingested by cancer cells gives them potential as novel efficient therapies that modulate the characteristics of the cancer cells through intercellular interactions.^16^, ^28^, ^29^, ^30^ Polyphenols are particularly promising candidates as oral administration of an aqueous extract of green tea, commonly known as catechin polyphenols, has recently been shown to inhibit UV radiation‐induced skin cancer in terms of tumor incidence, tumor multiplicity, and tumor growth/size. (−)‐Epigallocatechin‐3‐gallate (EGCG), a major catechin component, accounts for around 40–60% of the polyphenol content in green tea.^31^ Importantly, epidemiological studies have shown daily intragastric injection of EGCG could inhibit the progression and metastasis of ovarian cancer and prostate cancer in animal models.^32^

Polyphenols have also recently emerged as versatile building blocks for the engineering of functional particles and films.^33^ Metal‐phenolic networks (MPNs) composed of polyphenols and metals have been attracting great attention in the applications of biotechnology and biomedicine due to their high biocompatibility, versatile functionalization, and pH‐responsive disassembly.^34^, ^35^ Specifically, several metal ions including Fe^3+^, Pt^4+^, Cu^2+^, etc. have been integrated with MPN complexes for the engineering of self‐assembled nanoparticles for therapeutic functions. Inspired from these previous works,^36^, ^37^, ^38^, ^39^ we synthesized self‐assembled metal‐phenolic nanocomplexes simply comprising EGCG and functional lanthanide samarium ions (Sm^3+^) (**Figure** **1**). Sm^III^‐EGCG NPs can be internalized by cancer cells, degraded in acidic pH during endocytosis, and can thereby release the therapeutic building blocks of Sm^3+^ ions and EGCG. Through the process, Sm^3+^ ions chelated with polyphenols can simultaneously be delivered to the melanocytes and in turn enhance the therapeutic effects of polyphenols while bringing cohesion to EGCG to create a viable drug delivery system (Figure 1a). We found that Sm^III^‐EGCG suppresses melanoma proliferation and can significantly induce apoptosis in the B16F10 melanoma cell line through caspase‐3/7 and poly‐ADP‐ribose polymerase (PARP) activation. Our wound‐mimic melanoma migration experiments indicate that Sm^III^‐EGCG could efficiently inhibit the cell migration of melanoma in vitro, which is a key criterion for metastatic melanoma therapy. In vivo studies demonstrated that Sm^III^‐EGCG showed remarkable therapeutic effects on primary melanoma tumors and inhibition of metastasis from invading other organs.

**Figure 1:**
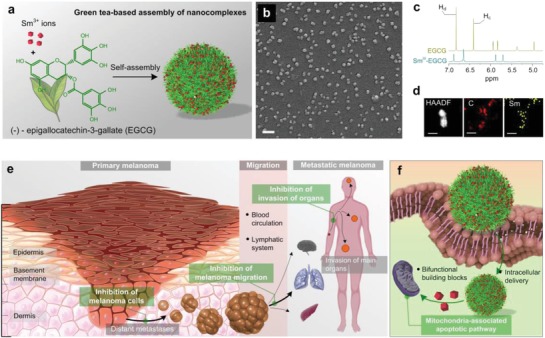
Green tea polyphenol‐based nanocomplexes and their therapeutic effects on metastatic melanoma. a) Facile synthesis of Sm^III^‐EGCG nanocomplexes through self‐assembly. b) SEM image of narrowly dispersed Sm^III^‐EGCG nanocomplexes with near‐spherical morphology. Scale bar is 100 nm. c) ^1^H NMR spectra of EGCG molecules and Sm^III^‐EGCG nanocomplexes demonstrate coordination complexation between the galloyl groups of EGCG and Sm^3+^ ions. d) HAADF‐TEM and EDS mapping images of Sm^III^‐EGCG nanocomplexes. Scale bars are 50 nm. e) Schematic illustration of the developments of metastatic melanoma, which transforms from primary melanoma and migrates to invade other organs. The administration of Sm^III^‐EGCG nanocomplexes can effectively inhibit the developments of metastatic melanoma through three main targeted therapeutic effects (green boxes). f) Internalization of Sm^III^‐EGCG into the melanoma cells, disassembly of green tea catechin and Sm^3+^ ions, and mitochondria‐associated apoptosis.

Sm^III^‐EGCG nanocomplexes were formed simply by mixing Sm^3+^ ions (4 mmol L^−1^) and EGCG (1 mmol L^−1^), followed by stirring for 24 h at neutral pH, resulting in a clear solution.^40^ Scanning electron microscopy (SEM) shows the nearly spherical morphology of the Sm^III^‐EGCG nanocomplexes with a hydrodynamic size distribution of 61.2 ± 2.1 nm (Figure 1b and Figure S1, Supporting Information). The zeta potential of hydrated Sm^III^‐EGCG was −36.8 ± 7.1 mV, which is more than a twofold shift (in magnitude) compared to our previously reported Fe^III^‐tannic acid (TA) coating (−18 ± 4 mV). The negative zeta potential was likely due to the phenolic building blocks, which is favorable for the dispersion of nanocomplexes in biological‐relevant environment and the efficient intracellular translocation of therapeutic compounds. Fourier‐transform infrared spectroscopy showed spectral changes between the Sm^III^‐EGCG and EGCG only. The skeletal vibration of the benzene ring at 1625−1440 cm^−1^ significantly reduced. The peaks of O—H in‐plane bending vibration and C—O stretching vibration at 1352 and 1226 cm^−1^ also showed significant decrease after the formation of nanocomplexes (Figure S2, Supporting Information). As shown in Figure 1c, the ^1^H NMR spectra of EGCG and Sm^III^‐EGCG in dimethyl sulfoxide‐*d*_6_ (DMSO‐*d*_6_) revealed signature peaks in the range of 5.5–7.0 ppm, Hd and Hc, with integral of two protons each, ranging from 6.90 to 6.25 ppm, are attributed to the galloyl's phenyl protons of EGCG. After chelating with Sm^3+^ ions, the peaks corresponding Hc and Hd and shifted downfield. This change could be attributed to the complexation of EGCG with metal ions as has been previously reported for the coordination of other galloyl groups.^39^ Furthermore, high‐angle annular dark field (HAADF) scanning transmission electron microscope (TEM) image reveals the individual nanostructure of Sm^III^‐EGCG nanocomplexes and the corresponding energy‐dispersive X‐ray spectroscopy (EDS) mapping images show the elemental distributions of coordinated Sm^III^ in the nanocomplexes (Figure 1d).

The effect of Sm^III^‐EGCG nanocomplexes on the proliferation of various cell lines was determined using a cholecystokinin‐octopeptide (CCK‐8) assay. The cell viability was examined for a melanoma cell line (B16F10 cells) and two normal cell lines (NIH3T3 cells and human lymphatic endothelial cells (HLECs)). Different cells lines were treated with Sm^III^, EGCG, and Sm^III^‐EGCG at varying concentrations from 1 to 250 µg mL^−1^ for 24 h. **Figure** **2**a demonstrates that Sm^III^‐EGCG significantly inhibited the proliferation of the B16F10 cells as the concentration increased. The cancer cell viability was 84.3% when the Sm^III^‐EGCG concentration was 1 µg mL^−1^ and greatly decreased to ≈39.7% when the Sm^III^‐EGCG concentration was 250 µg mL^−1^. Bright‐filed microscopy images also showed the decrease of cell numbers and morphological changes of B16F10 cells after the treatment of different concentrations of Sm^III^‐EGCG (Figure S3, Supporting Information). This is remarkable as the free Sm^III^ or EGCG potency values were individually lower than these values (≈77.5% of Sm^3+^ and ≈75.2% of EGCG only at 250 µg mL^−1^). This highlights the synergistic benefits of the nanocomplexation introduced herein. Importantly, Sm^III^‐EGCG had negligible effects on the normal healthy cell lines, with viabilities of ≈79% and ≈95% for NIH3T3 and HLEC cells, respectively (Figure 2b). Given that both of the building blocks are not anticancer drugs, the high specificity of the nanocomplexes to tumor shows as advantage compared with traditional chemotherapy.

**Figure 2:**
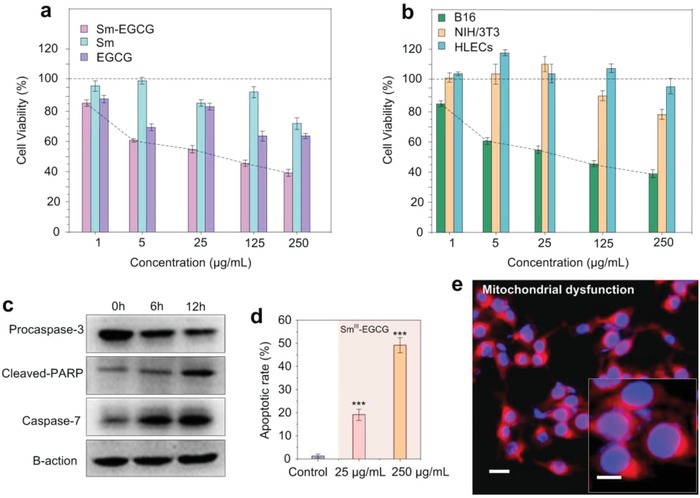
Selective toxicity of Sm^III^‐EGCG nanocomplexes on melanoma cells. a) Cell viability of a melanoma cell line (B16F10 cells) was determined by CCK‐8 assay after 24 h incubation with different concentrations of Sm^III^‐EGCG, EGCG, and Sm^3+^ ions individually. b) Cell viability of Sm^III^‐EGCG nanocomplexes on normal healthy cells (NIH/3T3 and HLECs cells) compared with B16F10 cells. c) Western‐blot analysis of mitochondria‐associated apoptotic protein expression after incubation with Sm^III^‐EGCG at different concentrations. d) Sm^III^‐EGCG‐induced apoptosis in B16F10 cells counted by using flow cytometry. The variation is represented by the standard deviation of three independent replicates in all graphs, ^***^ (*p*‐value < 0.05). e) Fluorescence microscopy images of generation of mitochondrial dysfunction. MitoTracker (red) probe indicates the change of mitochondrial membrane potential changes in B16F10 cells. 4′,6‐diamidino‐2‐phenylindole (DAPI) stain (blue) represents the cell nuclei. Scale bars are 20 and 10 µm in inset.

To determine the mechanisms by which Sm^III^‐EGCG nanocomplexes specifically inhibited melanoma cell proliferation, we performed western‐blot analysis of mitochondria‐associated apoptotic protein expression. Figure 2c shows that the level of procaspase‐3 decreased and caspase‐7 levels increased in B16F10 cell incubated with Sm^III^‐EGCG. Based on these results, it can be determined that upregulation of cleaved caspase‐3 leads to downregulation of procaspase‐3. It has been previously reported that caspase‐3 and caspase‐7 are the most efficient proteases for PARP cleavage.^41^ PARP is part of a family of proteins involved in a number of cellular processes that aid in DNA repair, DNA stability, and programmed cell death.^42^ This suggested that cleaved PARP was upregulated in B16F10 cells when the cells were incubated with Sm^III^‐EGCG. Based on these results, it can be rationalized that Sm^III^‐EGCG could trigger caspase‐3/7 activation and PARP cleavage in melanocytes, which ultimately results to the apoptosis of melanocytes. Flow cytometry further confirmed that Sm^III^‐EGCG induced apoptosis in B16F10 cells (Figure S4, Supporting Information). The apoptosis rate of B16F10 cells incubated with Sm^III^‐EGCG increased from 19.01% to 49.39%, while that of the control group was 0.42% (Figure 2d). Morphological changes in B16F10 cells were observed in the presence of Sm^III^‐EGCG (Figure S5, Supporting Information). Figure 2e and Figure S6 in the Supporting Information show the nanocomplex internalization, mitochondria membrane potential change, and dysfunction in B16F10 cells after treatment with Sm^III^‐EGCG nanocomplexes.

To determine if the melanoma cancer cell disruption translates into other beneficial inhibition of melanoma metastasis, we performed a wound‐induced migration assay (**Figure** **3**). After wounding, the major part of the wounded space between the cell layers was occupied by migrated melanoma cells when no treatment was performed. However, after treatment with the Sm^III^‐EGCG nanocomplexes, the cancer cell migration was severely inhibited in a concentration dependent manner. These findings suggested that Sm^III^‐EGCG nanocomplexes could efficiently inhibit the migration of melanoma cells, which solves one of the great challenges in metastatic melanoma therapy.^43^

**Figure 3:**
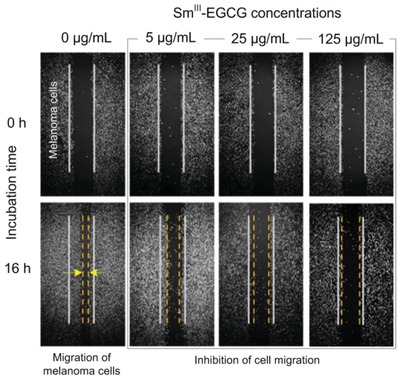
Wound‐induced migration assays indicated the inhibition of migration of B16F10 melanoma cells in the presence of Sm^III^‐EGCG nanocomplexes. In the control group, B16F10 melanoma cells expanded to the center area (yellow dashed lines) after 16 h due to the their migration capacity, which represents the most challenging point for the therapy of metastatic melanoma. After treatment with Sm^III^‐EGCG nanocomplexes, the presence of Sm^III^‐EGCG effectively inhibited the growth of B16F10 melanoma cells where the yellow dashed lines of 16 h migration were similar with the white solid lines of right after the wound was inflicted (0 h).

Sm^III^‐EGCG nanocomplexes were used to study the therapeutic effects on both subcutaneous melanoma and metastatic lung melanoma, respectively. We first validated the in vivo therapeutic effects of Sm^III^‐EGCG nanocomplexes on melanoma tumors using B16F10 cells. A melanoma xenograft model was created by injecting B16F10 cells into the flanks of immune‐deficient mice, wherein 5‐fluorouracil, a general clinic drug, was used as a positive control (**Figure** **4**a). As shown in Figure 4b–e, the tumor volume significantly decreased after treatment with the Sm^III^‐EGCG nanocomplexes. The average tumor size at 20 d of the group treated by Sm^III^‐EGCG nanocomplexes was 878.91 ± 71.14 mm^3^, which showed superior therapeutic effect over 5‐fluorouracil (1120.90 ± 117.32 mm^3^). While the untreated group showed a much larger tumor volume of 1750 ± 180 mm^3^. The body weights of the mice were monitored over the course of the treatment period (Figure 4f), and the 5‐fluorouracil‐treated group body weight obviously decreased compared with the control group; however, the body weight of the Sm^III^‐EGCG‐treated group showed no notable change. Moreover, these results suggested that Sm^III^‐EGCG exhibited significantly lower side effects than 5‐fluorouracil when the anticancer therapy was performed on melanoma primary tumors. The metabolic detailed mechanism of EGCG and Sm^3+^ ions possessing these targeting anticarcinogenic attributes is not clear in the literature, though their therapeutic efficiency is clear as is the case of our in vivo results. Further studies are required to elucidate the detailed mechanism of antitumor efficacy of these nanocomplexes.

**Figure 4:**
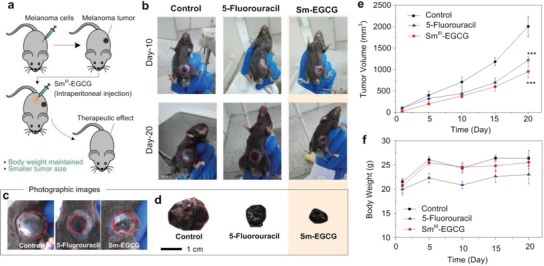
In vivo tumor therapy of Sm^III^‐EGCG nanocomplexes in a melanoma tumor model. a) Schematic of tumor formation and injection of Sm^III^‐EGCG nanocomplexes into the tumor. Mice were subcutaneously injected with 1.0 × 10^6^ B16F10 cells and the antitumor effects of 50 mg kg^−1^ Sm^III^‐EGCG or 5‐fluorouracil (30 mg kg^−1^) was evaluated. Saline was used as the control. b–d) Morphological changes in tumor size. c) Panel: Photographic images of mice bearing B16F10 tumors with treatments of PBS, 5‐fluorouracil, and Sm^III^‐EGCG. d) Panel: Photographic images of postmortem tumors. e) Tumor growth monitored at different time‐points and calculated tumor volume. The variation is represented by the standard deviation of three independent replicates in all graphs, ^***^ (*p*‐value < 0.05). f) Body weight per experimental group at the indicated time points. The variation is represented by the standard deviation of three independent replicates in all graphs.

We then examined the targeting potential of Sm^III^‐EGCG nanocomplexes to inhibit the metastatic spreading from melanoma in vivo. To do so, C57Bl/6J mice were inoculated intravenously with B16F10 cells and then were treated by intraperitoneal administrations with either saline or Sm^III^‐EGCG for 24 d (**Figure** **5**a). Figure 5b demonstrates several lung metastatic nodules in the control group and 5‐fluorouracil treated group; however, the treatment of Sm^III^‐EGCG led to relatively clear lung surfaces. Moreover, the group treated with Sm^III^‐EGCG nanocomplexes led to significantly lower numbers of metastatic nodules than 5‐fluorouracil‐treated group or control group (Figure 5c). The data indicated that Sm^III^‐EGCG had more merit as an antimetastasis agent with lower side effects compared with 5‐fluorouracil in vivo. We have performed experiments with an additional group of mice treated with vemurafenib as BRAF inhibitor (Figure S7, Supporting Information). The new in vivo results showed that vemurafenib could not inhibit the growth of B16 melanoma, probably because that the mouse melanoma cell line has no activated mutation in the 11 or 15 exon of the BRAF oncogene.^44^ This result suggested that our Sm^III^‐EGCG nanocomplex performed more general therapeutic effects on melanoma with no obvious selection to the gene biomarker. Moreover, histological results of the main organs, including heart, liver, kidney, and spleen, were further evaluated to determine the prolonged toxicity of the Sm^III^‐EGCG nanocomplexes. Compared with the untreated groups, the organs of the Sm^III^‐EGCG‐treated group had no obvious damage (Figure 5d). Collectively, these results were consistent with the in vitro results that Sm^III^‐EGCG nanocomplexes selectively and effectively induce the apoptosis of tumor cells without negative side effects on normal healthy cells.

**Figure 5:**
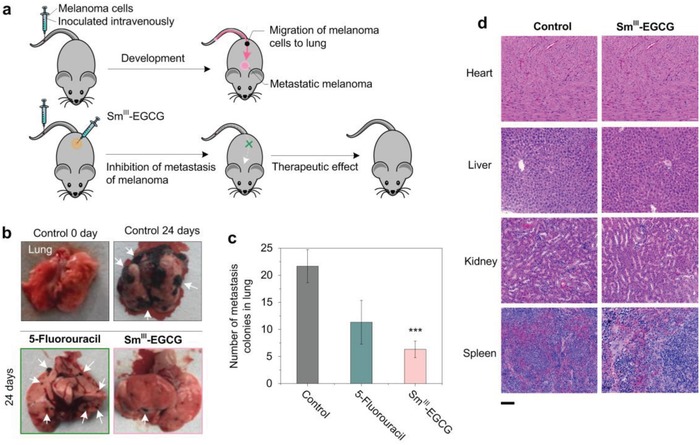
Sm^III^‐EGCG nanocomplexes were successful in treating a metastatic lung melanoma model. a) Schematic of metastatic melanoma formation and inhibition by Sm^III^‐EGCG nanocomplexes. b) Morphological changes in lungs at 0 and 24 d. In the control and 5‐fluorouracil‐treated groups, black dots can be seen on the lung, while no obvious black dots can be observed in the Sm^III^‐EGCG‐treated group. c) Static numbers of metastatic nodules in different groups. The variation is represented by the standard deviation of three independent replicates in all graphs, ^***^ (*p*‐value < 0.05). d) Histopathology of the heart, liver, and kidney from mice. The main organs were collected and processed for histological analysis. The sections were stained with hematoxylineosin. Images are representative of three independent experiments. The scale bar is 100 µm.

In summary, we have developed and characterized a green tea‐based MPN nanocomplex engineered from natural phenolics and lanthanide ions. This system was formulated by the simple self‐assembly of EGCG molecules and lanthanide Sm^3+^ ions. The combined therapeutic effects of the green tea‐based EGCG building block and the lanthanide ions showed greater anticancer effects on melanoma cells than the individual components and generated the targeted cell apoptosis of tumor cells through mitochondrial dysfunction. Sm^III^‐EGCG nanocomplexes effectively inhibited the migration of melanoma cells in a chip‐based experimental setting. Similarly, in vivo results revealed that the administration of Sm^III^‐EGCG dramatically decreased the tumor volume and metastasis of melanoma through targeted therapeutic effects with no obvious systemic toxicity. Our results suggest that these green tea‐based nanocomplexes meet the key criteria of a clinically promising treatment for challenging advanced stage melanoma, and further research on the clinical application of Sm^III^‐EGCG nanocomplexes is ongoing.

## Conflict of Interest

The authors declare no conflict of interest.

## Supporting information

Supplementary
