# Supplementary material for: Targeted Therapy against Metastatic Melanoma Based on Self‐Assembled Metal‐Phenolic Nanocomplexes Comprised of Green Tea Catechin
Source: Adv Sci (Weinh). 2019 Jan 15;6(5):1801688. doi: 10.1002/advs.201801688 (PMC6402403; doi:10.1002/advs.201801688)
Supplement: Supplementary file 1 — Supplementary [file ADVS-6-1801688-s001.pdf]

Copyright WILEY-VCH Verlag GmbH & Co. KGaA, 69469 Weinheim, Germany, 2019.

ADVANCED SCIENCE
Open Access

Supporting Information

for *Adv. Sci.,* DOI: 10.1002/advs.201801688

Targeted Therapy against Metastatic Melanoma Based on Self-Assembled Metal-Phenolic Nanocomplexes Comprised of Green Tea Catechin

Ke Li, Gao Xiao, Joseph J. Richardson, Blaise L. Tardy, Hirotaka Ejima, Wen Huang, Junling Guo, Xuepin Liao,* and Bi Shi*

Supplementary Information

Targeted therapy against metastatic melanoma based on self-assembled metal-phenolic nanocomplexes comprised of green tea catechin

Ke Li[1,2], Gao Xiao[3,4], Joseph J. Richardson[5], Blaise L. Tardy[6], Hirotaka Ejima[7], Wen Huang[2], Junling Guo[1,3,8,], Xuepin Liao[1,*], Bi Shi[1,8]*

[1]Department of Biomass Chemistry and Engineering, Sichuan University, Chengdu 610065, China.

[2]Laboratory of Ethnopharmacology, Regenerative Medicine Research Center, West China Hospital, Sichuan University, Chengdu, Sichuan 610041, China.

[3]Wyss Institute for Biologically Inspired Engineering, John A. Paulson School of Engineering and Applied Sciences, Harvard University, Boston, Massachusetts 02115, United States.

[4]Department of Environmental Science and Engineering, College of Environment and Resources, Fuzhou University, Fuzhou 350108, China.

[5]ARC Centre of Excellence in Convergent Bio-Nano Science and Technology and Department of Chemical and Biomolecular Engineering, The University of Melbourne, Parkville, Victoria 3010, Australia.

[6]Department of Bioproducts and Biosystems, School of Chemical Engineering, Aalto University, P. O. Box 16300, 00076, Finland.

[7]Department of Materials Engineering, The University of Tokyo, 7-3-1 Hongo, Bunkyo-ku, Tokyo 113-8656, Japan.

[8]National Engineering Laboratory for Clean Technology of Leather Manufacture, Sichuan University, Chengdu, Sichuan 610065, China.

**Correspondence to: junling.guo@scu.edu.cn, junling.guo@wyss.harvard.edu (J.G.); xpliao@scu.edu.cn (X.L.)*

Table of Contents

Section 1. Preparation of Complexes....3
Section 2. Cell Culture....3
Section 3. Cell Proliferation Assay....3
Section 4. Cell Apoptosis Detection....4
Section 5. Western Blotting....4
Section 6. Transwell Migration Assay....5
Section 7. Experimental Melanoma Tumor Model....5
Section 8. Experimental Melanoma Metastasis Model....6
Section 9. Statistical Analysis....6
Section 10. Supplementary Tables and Figures....7

Section 1. Preparation of Complexes

In optimal conditions, Sm(III) ($Sm(NO_3)_3.6H_2O$) and EGCG in a molar proportion (Sm: EGCG) of 4: 1 were dissolved in EtOH (40 mL) to obtain final concentrations of 4 mmol/L and 1 mmol/L, and the mixture was stirred until the solid was dissolved. The mixture was adjusted by sodium carbonate solution to pH 7.0 and reacted at 37 °C for 24 h. Then, the complex in the mixture was harvested by centrifugation and washed several times with ethanol, and finally the Sm^{III}-EGCG complex with brown color was obtained after vacuum drying. The as prepared Sm^{III}-EGCG was identified by ^{1}H NMR spectra (Bruker Avance 500 MHz NMR Spectrometer, USA) analysis.

Section 2. Cell Culture

B16F10 cells (Murine melanoma cell line), NIH3T3 cells (fibroblast cell line), HUVEC cells (Human Umbilical Vein cell line), HLECs cells (Human lens epithelium cell line) were recipient from Lab of Transplant Engineering and Immunology, Regenerative Medicine Research Center, West China Hospital. The NIH3T3, HUVEC and HLECs cell lines were cultured in DMEM, the B16F10 cell line was maintained in RPMI-1640 medium supplemented with 10% fetal bovine serum (FBS), penicillin (100 U/mL) and streptomycin (100 mg/mL). Cells were sustained at 37 °C in a humidified atmosphere with 5% CO_2.

Section 3. Cell Proliferation Assay

Cell proliferation was analyzed using Cell Counting Assay Kit-8 (CCK-8) (Dojindo Molecular Technologies, Japan). 100 μL of cell suspension (5×10^3 cells/well) was dispensed into a 96-well plate and pre-incubated overnight, then exposed to various concentrations of Sm^{III}-EGCG for 24 h. Ten microliters of CCK-8 solution was added to each well. After incubation for 1 h, the absorbance at 450 nm was measured using microplate reader. Experiments were performed at least three times with

representative data presented.

Section 4. Cell Apoptosis Detection

Cell apoptosis analysis was performed using Annexin V/PI apopotosis assay kit (KeyGEN Biotech, China). Various concentrations of Sm^{III}-EGCG for 24 h, melanoma cells were collected by centrifugation and resuspended in 500 µl of binding buffer. Then, 5 µl of Annexin V-FITC was added to the re-suspended cells. After incubation for 5 min on ice in the dark, 1 µg of PI was added to the cell suspension. Apoptotic and necrotic cells were quantified using a FACScan flow cytometer and the Cell Quest pro software (Beckton-Dickinson).

Section 5. Western Blotting

B16F10 cells in 6-well plate were collected and washed with PBS and then lysed with 60 µL lysis buffer (Beyotime) for 15 min at 4 °C. The cell supernatant was collected by centrifugation at 12,000 g centrifuged for 10min at 4 °C. Protein concentration was determined using the BCA protein assay kit. The equal amount (30 µg) of extract protein was loaded, separated by 15% SDS-PAGE, and transferred to a polyvinylidene difluoride membrane (PVDF, Carlo Erba reagents, Milan, Italy). The transferred membranes were blocked with 5% skim milk in Tris-buffered saline- Tween 20 (TBST: 0.1% Tween 20, 100 mM NaCl and 10 mM Tris- HCl, (pH 7.6)) for 2 h at room temperature. After blocked with 5% milk in the TBST buffer for 1 h, membranes were subsequently probed with appropriate primary antibodies overnight at 4 °C. The membranes were then incubated with HRP-conjugated secondary antibodies and analyzed by using ChemiDoc™- XRS imaging system (Bio-Rad). Quantifications of relative protein expression were carried out were performed with the Image Lab 3.0 software (Bio-Rad).

Section 6. Transwell Migration Assay

Transwell migration assay was performed to assess the effect of Sm^{III}-EGCG on melanoma cell migration as previously described (24). Wounds were created by scratching the cells using a plastic pipette tip, and any loose cellular debris or detached cells were removed by PBS wash. The medium was replaced with fresh medium 24 h. The wound gap was observed and cells were photographed using phase-contrast microscopy. The gaps of the wounds were observed with phase contrast microscopy and digitally photographed. Each experiment was performed in triplicate.

Section 7. Experimental Melanoma Tumor Model

Male C57Bl/6J mice (4 weeks old) with body weights ranging from 18-20 g were purchased from Chengdu Dossy experimental animal Co. Ltd. (Chengdu, China). The animal room was controlled to maintain temperature (22 ± 2°C), light (12 h light/dark cycles) and humidity (50 ± 10%). After 1 week of acclimatization, the mice were depilated and implanted in the right flank region with the B16F10 cells (1.0×10^6). Seven days following implantation, the mice with tumor sizes > 50 mm^3 were selected and randomly divided into three groups (n = 10 per group), termed the model, Sm^{III}-EGCG and 5-FU groups. These groups were treated with saline, 150 mg/kg Sm^{III}-EGCG and 30 mg/kg 5-FU respectively, the concentrations of which were determined in a preliminary study. Equal volumes of the drugs and vehicle were administered orally to the mice every day for 3 weeks. The body weight and tumor size were measured three times weekly and the tumor volumes were calculated according to the following formula: $width^2 \times length \times 0.5$. All experimental animals were treated according to the protocols approved by the Bioethics Committee of West China Center of Medical Sciences, Sichuan University.

Section 8. Experimental Melanoma Metastasis Model

40 male C57Bl/6J mice injected intravenously via the tail vein with 1 × 10^6 B16F10 cells to produce experimental lung metastasis after 1 week of acclimatization. Intraperitoneal is herein preferred as its enables a maintained bioavailability and monodispersity compared to intravenous injection. Although, ultimately intravenous injection may yield to a final clinical use method, intraperitoneal injections are still used in this work because of the center on fundamental study and clinical fact that many antitumor drugs, including BARF inhibitors, are administered intragastrically. The mice were randomly divided into four groups (n=10): vehicle group, model group, Sm^{III}-EGCG group and 5-FU (positive vehicle) group. Animals in each group were intraperitoneally injected with Sm^{III}-EGCG 150 mg/kg, 5-FU 30 mg/kg or saline as vehicle once daily. All animals were scarified on the 24th day, followed by a comprehensive visual examination of all organs. Black dots on lung surface were counted and confirmed as melanoma metastases. Mouse tissue samples (heart, liver, spleen and kidney) were fixed in 4% paraformaldehyde and embedded in paraffin. The tissue samples were processed into sections of 4 μm thick sections and the slides were stained with hematoxylin and eosin (H&E) according to standard protocols.

Section 9. Statistical Analysis

Data were expressed as mean ± standard deviation (*SD*). Data were analyzed with one-way ANOVA using SPSS 16.0. Differences were considered statistically significant when $P < 0.05$.

Section 10. Supplementary Tables and Figures

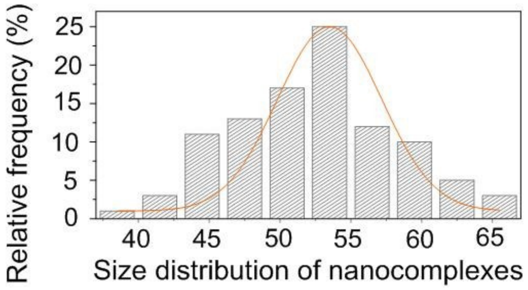

Figure S1. Size distribution of Sm^{III}-EGCG nanocomplexes (54.5 ± 5.3 nm) measured by SEM. More than 100 particles were measured. Hydrodynamic size distribution of 61.2 ± 2.1 nm was obtained by DLS measurement which agrees with the measured result based on SEM images. This result suggests the monodispersity of nanocomplexes without significant aggregation in hydrated suspension.

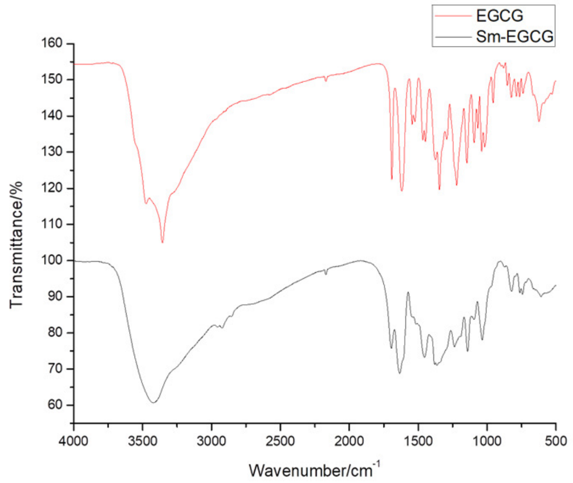

Figure S2. FTIR spectra of Sm^{III}-EGCG nanocomplexes and EGCG only.

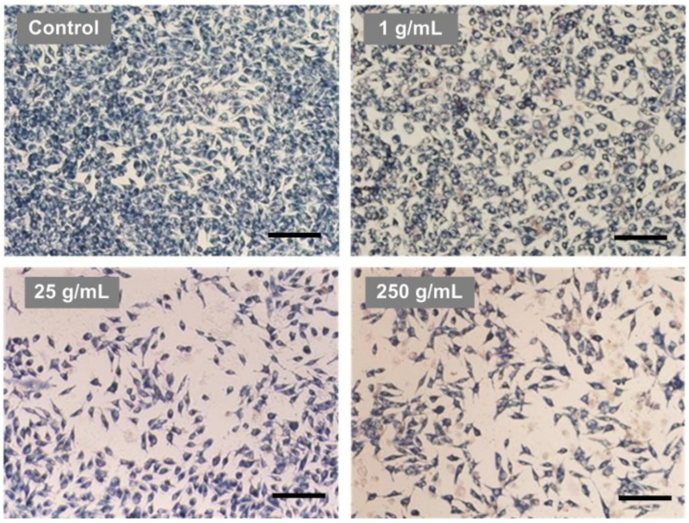

Figure S3. Brightfield microscopy images of B16F10 cells after with the treament with various concentrations of Sm^{III}-EGCG for 24 h. Scale bar are 100 μm.

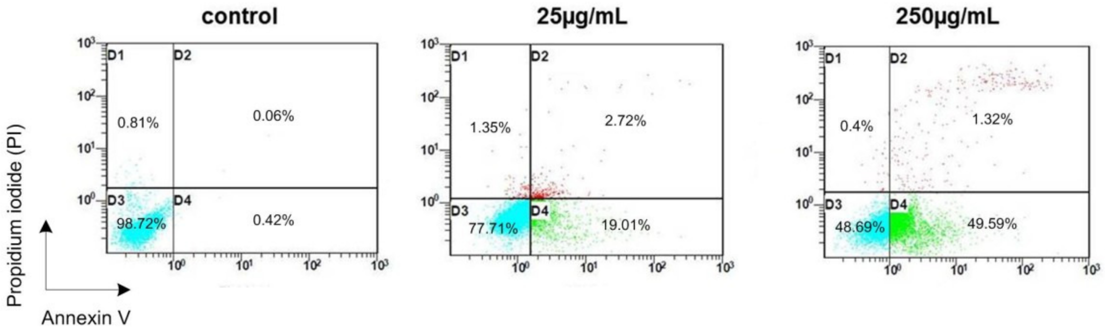

Figure S4. The apoptotic effect of Sm^{III}-EGCG on B16F10 cells. Quadrants of (a) control group and treated groups with Sm^{III}-EGCG concentrations of (b) 25 μg/mL and (c) 250 μg/mL. The apoptotic cells were measured by FACS analysis after Annexin V and PI staining.

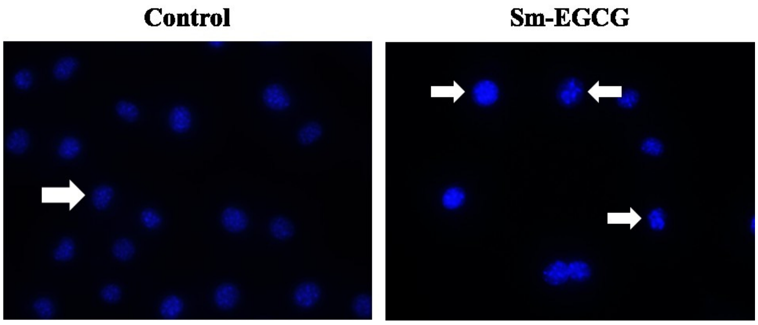

Figure S5. Morphologic changes in B16F10 cells in the presence of Sm^{III}-EGCG were examined by using Hoechst 33258 fluorescence staining.

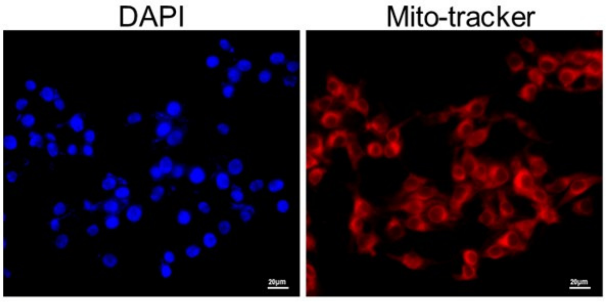

Figure S6. Co-localization of Mito-Tracker (red) in B16 cells. Cells were incubated in 250μg/mL Sm^{III}-EGCG for 24 h and stained with Mito-Tracker. DAPI (blue) was used for nuclei detection. The images are representative of 3 independent experiments. Scale bars are 20 μm.

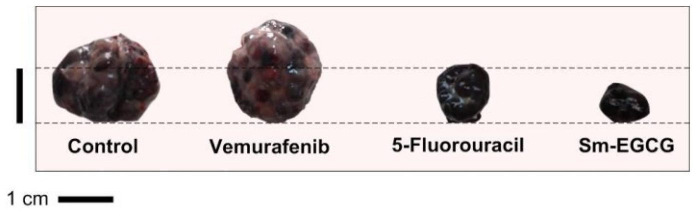

Figure S7. **Photographic images of post-mortem tumors show the morphological changes in tumour size at 20 days of the each group**. Vemurafenib was used as BRAF inhibitor for the additional control group of melanoma treatment. The result suggested that BRAF inhibitor-based drug does not show inhibition on C57 mice inoculated with B16 cells. Scale bar is 1 cm.
